# Brain microstructural alterations of depression in Parkinson's disease: A systematic review of diffusion tensor imaging studies

**DOI:** 10.1002/hbm.26015

**Published:** 2022-07-20

**Authors:** Mohammad Amin Salehi, Soheil Mohammadi, Mahdi Gouravani, Arian Javidi, Stephen R. Dager

**Affiliations:** ^1^ School of Medicine Tehran University of Medical Sciences Tehran Iran; ^2^ Department of Radiology University of Washington Seattle Washington USA

**Keywords:** cognitive impairment, depression, diffusion tensor imaging, Parkinson disease, systematic review

## Abstract

Depression, a leading cause of disability worldwide, is also the most prevalent psychiatric problem among Parkinson disease patients. Both depression and Parkinson disease are associated with microstructural anomalies in the brain. Diffusion tensor imaging techniques have been developed to characterize the abnormalities in cerebral tissue. We included 11 studies investigating brain microstructural abnormalities in depressed Parkinson's disease patients. The included studies found alterations to essential brain structural networks, including impaired network integrity for specific cortical regions, such as the temporal and frontal cortices. Additionally, findings indicate that microstructural changes in specific limbic structures, such as the prefronto‐temporal regions and connecting white matter pathways, are altered in depressed Parkinson's disease compared to non‐depressed Parkinson's disease and healthy controls. There remain inconsistencies between studies reporting DTI measures and depression severity in Parkinson disease participants. Additional research evaluating underlying neurobiological relationships between major depression, depressed Parkinson's disease, and non‐depressed Parkinson's disease is required to disentangle further mechanisms that underlie depression and related somatic symptoms, in Parkinson disease.

## Graphical abstract

We provide a systematic review of current literature on DTI in DPD subjects. DPD patients show microstructural abnormalities in white matter integrity and essential structural networks of the brain. Affected white matter is mostly found in the thalamus, temporal, and frontal cortices.

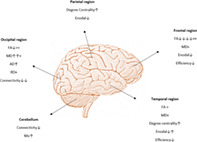

## 1 INTRODUCTION

Parkinson disease (PD) is a progressive neurodegenerative disorder exhibiting a complex set of clinical features, comprising both nonmotor symptoms (NMS), including cognitive dysfunction, psychosis, and mood disorders, and motor features including the cardinal triad of tremor, rigidity, and bradykinesia (Langston, 2006). As the most significant risk factor in the development of PD is aging, the increasing proportion of the elderly in the global population have made this condition the fastest‐growing neurologic cause of disability (GBD 2016 Neurology Collaborators, 2018). Moreover, depression, a leading cause of disability worldwide, is also the most prevalent psychiatric problem among PD patients, substantially worsening their quality of life (Aarsland et al., 1999; Tandberg et al., 1996). Although depressive symptoms, such as sadness and anhedonia, occur in nearly half of the patients with PD (Gotham et al., 1986), the identification of these features is a clinically challenging task since psychomotor and somatic presentations of depression can highly resemble the typical hallmarks of PD, such as bradykinesia and hypomimia, as well as decreased concentration and appetite. Thus, depressed PD (DPD) patients are at risk of delayed diagnosis and under treatment (Schrag, 2006).

It is well documented that the development of mood disturbances can precede motor manifestations in PD cases, underscoring the fact that associated depression does not emerge only secondary to the disabilities caused by PD but possibly shares similar pathophysiology (Ishihara & Brayne, 2006). While the exact pathogenesis of PD remains unclear, the accumulation of Lewi bodies, mainly containing α‐synuclein protein, as well as mitochondrial dysfunction and defective proteolysis, in regions such as the substantia nigra, are considered to be possible causes of neuronal cell death and the resultant reduction of striatal dopamine levels observed in PD brains (Calo et al., 2016; Lim & Zhang, 2013; Shulman et al., 2001; Wakabayashi et al., 2007). In this regard, neuroimaging techniques can provide invaluable insights into biochemical, structural, and functional alterations in the brains of DPD patients paving the way for the development of more precise and objective diagnostic and prognostic criteria in research and clinical settings (Won et al., 2019). Positron emission tomography (PET) and single‐photon emission tomography (SPECT) studies demonstrate metabolic changes in the striatum, limbic thalamus, and frontal lobe, involving dopaminergic and serotonergic pathways (Chagas et al., 2013; Weintraub et al., 2005). Likewise, magnetic resonance imaging (MRI) studies of DPD demonstrate gray matter (GM) and white matter (WM) volume alterations (Scheuerecker et al., 2010; Wen et al., 2016). Research has also identified altered brain networks involving subcortical, frontolimbic, and corticocortical fibers of DPD patients compared to non‐depressed PD (NDPD) patients (Ansari et al., 2019; van Mierlo et al., 2015).

More recently, diffusion tensor imaging (DTI) techniques, or diffusion‐weighted imaging (DWI), have been developed to characterize alterations in the microstructure and integrity of WM fiber tracts in psychiatric and neurological disorders, including PD (Basser et al., 1994; McKinstry et al., 1992). DTI measures the size and direction of water molecules' diffusion modeled using a voxel‐based three‐dimensional Gaussian plot distribution (Sanjari Moghaddam et al., 2019; van Ewijk et al., 2012). In the absence of any hindrance, the diffusion coefficient will be equal (isotropic) in all directions, as in GM and pure water. However, the presence of myelin sheath and cellular membranes in the WM shall act as barriers; hence this coefficient differs in each direction (anisotropic), with maximum diffusion along the axon bundles and minimum diffusion perpendicular to the axon (Beaulieu, 2002). Two main DTI indexes indicating WM tracts' organization and direction and myelination are mean diffusivity (MD) and fractional anisotropy (FA). As a mean of the water molecules' diffusion coefficients in primary directions, MD is higher in free extracellular spaces reflecting the easier movement of water in these areas regardless of the direction and lower in an intact WM due to limited free diffusion (Tievsky et al., 1999).

On the other hand, the FA index represents the directionality of water molecules ranging from zero (completely isotropic diffusion) to one (completely anisotropic diffusion). Unlike MD, decreased FA generally indicates diminished WM integrity (Alexander et al., 2007). Two other DTI metrics, axial diffusivity (AD) and radial diffusivity (RD) quantify water diffusion in the direction of nerve fibers and perpendicular to them, respectively. Although AD is generally considered to be an indicator of axonal integrity, and RD an indicator of myelin sheath intactness, the interpretation of these measurements in neurological disorders should be made cautiously as other parameters, such as axonal diameter and density, can influence these indices (Sanjari Moghaddam et al., 2019; Wheeler‐Kingshott & Cercignani, 2009).

Diffusion tensor imaging measures are robust biomarkers for progression in early‐stage PD and potential biomarkers for other stages of this disease (Mitchell et al., 2021); however, inconsistent DTI findings have been reported in relation to symptom severity of DPD patients. Whereas several studies have reported decreased FA in the group of WM tracts located in the thalamic, limbic, frontal, and cerebellar areas (Ghazi Sherbaf et al., 2018; Matsui et al., 2007; Wu et al., 2018), other studies demonstrate no relationships between DTI indices and depression symptoms and an absence of specific changes in WM integrity of DPD patients compared with NDPD individuals (Lacey et al., 2019; Surdhar et al., 2012). Considering the potential clinical and research utility of DTI measures for the early assessment of patients with PD and depressive disorders, we conducted the current review. The intent was to systemically tabulate and assess the results of DTI studies that specifically compared white matter microstructure and white matter tracts between DPD patients, NDPD patients, and healthy controls (HC), and possible relationships to clinical measures of depression.

## 2 METHODS

### 2.1 Study selection and data extraction

Preferred Reporting Items for Systematic Reviews and Meta‐analysis (PRISMA) guidelines were followed to perform the current analysis (JPT et al., 2020); the study protocol was designed and registered at the International Prospective Register of Systematic Reviews (PROSPERO) website (Registration No. CRD42021258388). We searched PubMed and EMBASE databases using a combination of keywords ((("Diffusion Tensor Imaging" OR “diffusion weighted magnetic resonance imaging” OR “Diffusion Tractography” OR “diffusion weighted imaging” OR “diffusion weighted MRI” OR “DTI” OR “white matter” OR “grey matter” OR “gray matter”)) AND ((“Parkinson Disease” OR “Idiopathic Parkinson's Disease” OR “Lewy Body Parkinson's Disease” OR “Parkinson's Disease, Idiopathic” OR “Parkinson's Disease, Lewy Body” OR “Parkinson Disease, Idiopathic” OR “Parkinson's Disease” OR “Idiopathic Parkinson Disease” OR “Lewy Body Parkinson Disease” OR “Primary Parkinsonism” OR “Parkinsonism, Primary” OR “Paralysis Agitans”))) AND ((“Depression” OR “Depressive Symptoms” OR “Depressive Symptom” OR “Symptom, Depressive” OR “Symptoms, Depressive” OR “Emotional Depression” OR “Depression, Emotional” OR “Depressions, Emotional” OR “Emotional Depressions”)) to identify possibly relevant literature published up to June 2021. We included all case–control, cohort, and cross‐sectional human studies examining DPD patients for WM microstructure changes in DTI measurements. Full‐text manuscripts were available regardless of their publication date, geographic location, and participants' age group. Animal studies, conference abstracts, non‐English articles, non‐original literature including book chapters, reviews, case studies, and letters were excluded.

Comprehensive review of the manuscripts entailed the extraction of the following data from the included studies: first author's name, publication year and location, number of DPD, NDPD, and HC subjects, demographic and medical data of participants, including age, sex, PD duration, depressive state, Parkinson medication, other medications, and common neurological findings, imaging parameters that included DTI field strength and DTI analysis method, and between‐group DTI findings.

### 2.2 Quality assessment

The quality of the included studies was evaluated by two reviewers (AJ and MAS) using the Newcastle‐Ottawa scale (NOS) for nonrandomized studies, which assesses three methodological quality aspects of sample selection, case and control groups' comparability, and exposure determination method with a maximum score of four, two, and two for each domain, respectively (Stang, 2010; Table S1). Moreover, the risk of publication bias for each study was appraised through criteria developed by Viswanathan et al. in a design‐specific manner (Viswanathan et al., 2008; Table S2). A third reviewer (SM) was consulted in case of any discrepancies in this process.

## 3 RESULTS

### 3.1 Summary of reviewed studies

**FIGURE 1:**
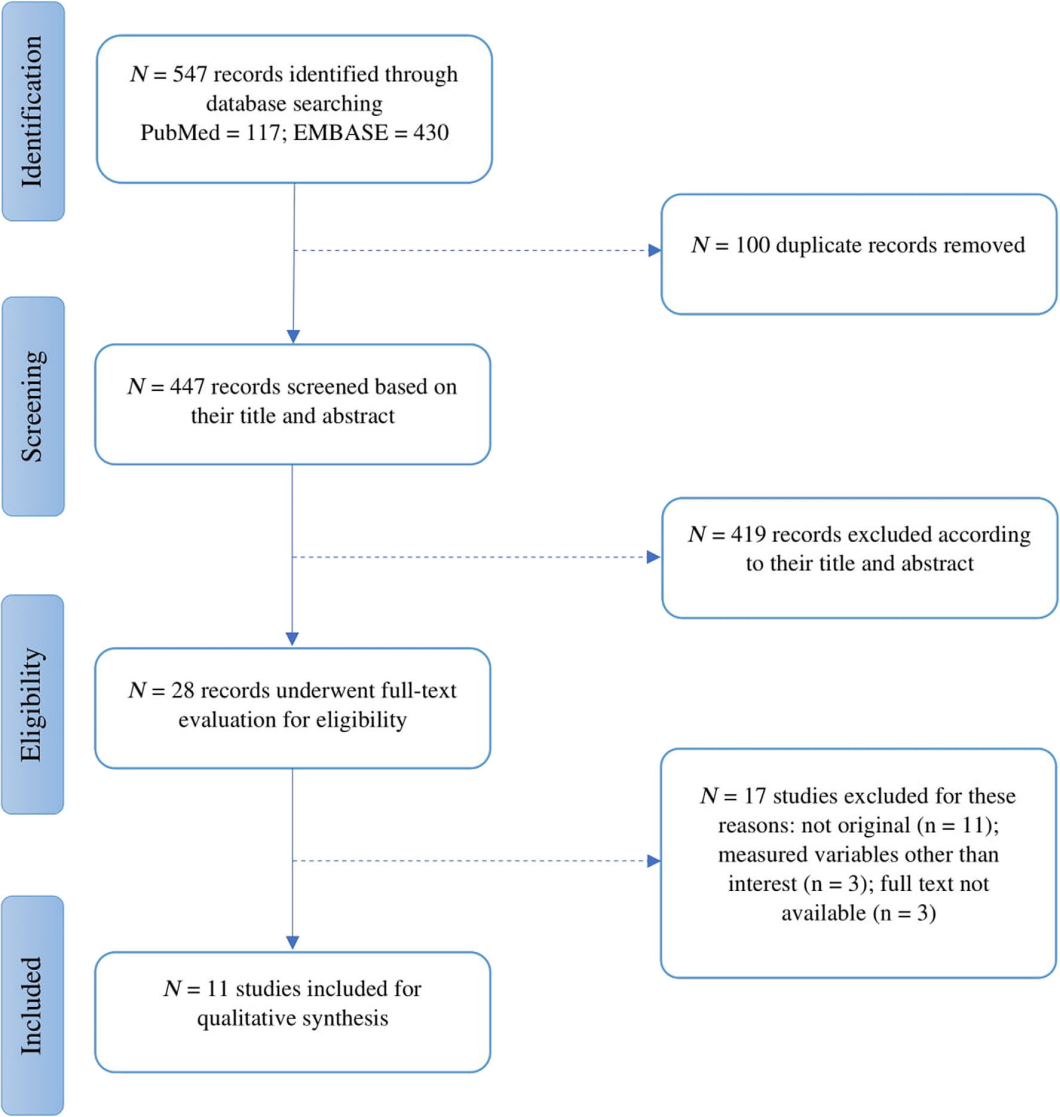
Flow diagram of the study selection

The search of databases yielded 547 articles. After automated and manual removal of duplicate records from search results (*n* = 100), the remaining articles underwent screening by two authors (AJ and MAS) based on their title or abstract, which resulted in the exclusion of an additional 419 records out of 447. Finally, 17 additional studies were excluded in the full‐text screening based on: not being original research (*n* = 11); measured variables other than those of interest (*n* = 3); unavailability of full‐text (*n* = 3). In the next phase, backward reference screening was also conducted on primarily included records to detect potentially missed eligible articles. Conflicts that emerged in this stage were resolved through discussion. Figure 1 illustrates the complete search and screening process resulting in the final selection of 11 studies for qualitative synthesis.

**FIGURE 2:**
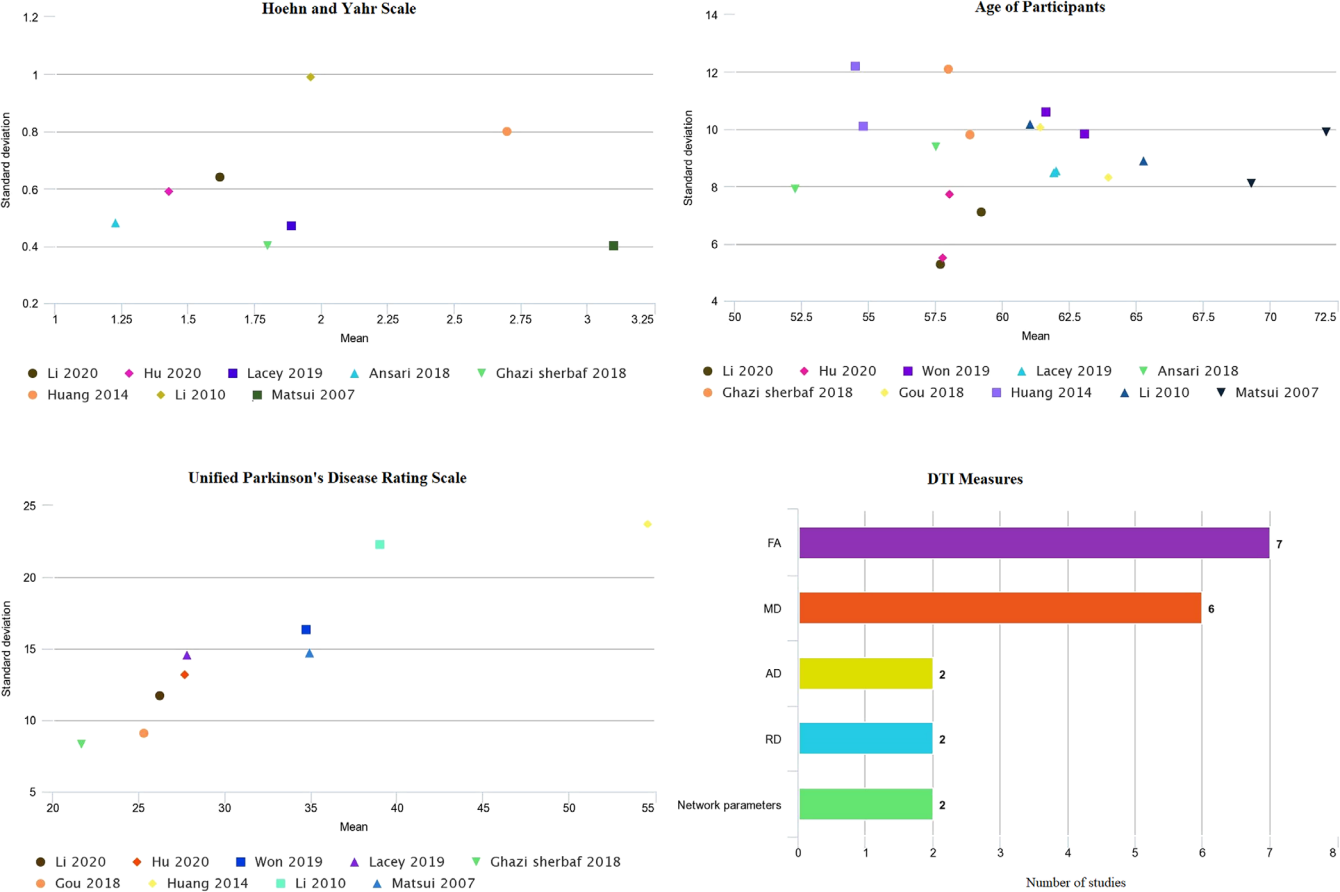
Age of participants for each study, both cases and controls, PD scale scores of study participants, and overview of DTI measures in included studies

Eleven DTI studies were extracted in total. All studies were case–control studies except for the study by Won et al., which was a retrospective cohort (Won et al., 2019). Diagnosis of DPD and NDPD in studied patients was verified in all studies. The severity of depression in DPD was assessed using well‐established mood rating scales, including the Hamilton Depression Rating Scale (HDRS; Hamilton, 1960), the Beck's Depression Inventory (BDI; Beck et al., 1960), and Geriatric Depression Scale (GDS; Meer & Baker, 1966). In addition to these mood rating scales, the studies of Li et al., Hu et al., and Huang et al. had an experienced psychiatrist diagnose depression using the Diagnostic and Statistical Manual of Mental Disorders (DSM) criteria (Hu et al., 2020; Huang et al., 2014; Li et al., 2020). In the Huang et al. study, depression was diagnosed as post‐morbid to PD (Wen et al., 2016). Studies extracted all confirmed DPD subjects and separated DPD subjects from NDPD subjects. Study demographics and participants' clinical data are summarized in Table 1. Dissemination of key features, such as age and PD scale scores, can be seen in (Figure 2). Six studies were conducted in China from 2018 onwards (Gou et al., 2018; Hu et al., 2020; Huang et al., 2014; Li et al., 2010, 2020; Won et al., 2019). Subjects matched for age and sex were used in all included studies except for the study by Prange et al., which matched study subjects just for age, as well as the studies of Ansari et al. and Matsui et al. in which cases and controls were not matched (Ansari et al., 2019; Matsui et al., 2007; Prange et al., 2019). Disease duration was more than 5 years in all studies that reported this parameter. The mean age of participants in all studies was more than 50 years.

**TABLE 1:** Overview of included studies; demographic and subject characteristics

Study		Study groups (exclusion criteria/particular distinctions)	Parkinson's disease diagnostic criteria	Depression diagnostic criteria	*N*(DTI^a^)/male	Age (years) (mean ± SD)	PD duration (years ± SD)	PD medication	Depression treatment	Matched for	Differed in
*Diffusion studies*											
1	Li/2020	DPD NDPD HC	UKPDSBB criteria used for idiopathic PD	DSM‐V and HDRS‐17	30/13 43/24 91/40	59.23 ± 7.10 58.09 ± 6.52 57.67 ± 5.27	5.46 ± 4.25 6.28 ± 3.38 –	None None –	None None –	Age, gender, years of education, UPDRS III scores, H&Y scores, PD duration	HDRS values
2	Won/2019	DPD NDPD	MDS‐UPDRS	GDS	36/24 45/30	61.63 ± 10.60 63.08 ± 9.83	– –	– –	– –	Age, sex, MDS‐UPDRS	GDS value
3	Prange/2019	Apathetic Parkinson patients Non‐apathetic PD patients HC	MDS‐UPDRS	LARS, SAS (for apathy), BDI‐II(for depression)	14/11 13/8 15/9	62.5 54 55	– = –	– – –	– – –	Age	LARS score, SAS score, BDI‐II score
4	Lacey/2019	DPD NDPD	–	GDS‐15	18/11 19/11	61.94 ± 8.48 62.00 ± 8.54	6.89 ± 0.96 6.74 ± 0.93	– –	Depression: 1 treated/17 never treated NDPD: No lifetime treatment	Age, sex, education level, MoCA, UPDRS—III, H&Y stages, freezing of gait, duration of PD	GDS value and H&Y stages
5	Ansari/2018	DPD NDPD	MDS‐UPDRS	GDS‐15	40/21 19/10	57.28 ± 7.9 57.5 ± 9.38	– –	– –	– –	–	–
6	Ghazi Sherbaf/2018	Depression+/RBD+ PD Depression+/RBD− PD Depression−/RBD– PD Depression−/RBD+ PD HC	MDS‐UPDRS	GDS‐15	14/3 43/19 20/7 16/4 31/13	58.8 ± 9.8 58.5 ± 8.7 58.4 ± 9.4 59.2 ± 11.6 58.0 ± 12.1	7.5 ± 7.5 6.2 ± 6.7 6.3 ± 6.8 8.5 ± 7.5 –	– – – –	– – – –	Age, sex	GDS and RBD score
7	Huang/2014	DPD NDPD	UKPDSBB	DSM‐IV	15/9 15/9	54.5 ± 12.2 54.8 ± 10.1	5.3 ± 4.8 4.2 ± 4.0	Anti‐Parkinsonian medicine was terminated at least 12 h prior to the imaging scans.	None None	Age, gender, education, disease duration, H&Y stages, side‐of‐onset, UPDRS scores, MMSE scores	HRSD score
8	Li/2010	DPD NDPD	UKPDSBB	DSM‐IV	14/4 18/10	65.28 8.89 61.05 10.17	6.29 ± 5.51 5.67 ± 2.57	– –	None None	Age, sex, PD duration time, MMSE, UPDRS, H&Y, and LEDD	HAMD score
9	Matsui/2007	DPD NDPD	UKPDSBB	DSM‐IV	14/2 14/4	72.1 ± 9.9 69.3 ± 8.1	8.8 ± 5.2 7.4 ± 5.1	– –	– –	H&Y stage	–
*Network studies*											
1	Hu/2020	DPD NDPD HC	UKPDSBB criteria for idiopathic PD	DSM‐V and HDRS‐17	20/9 47/25 46/22	58.05 ± 7.72 57.94 ± 6.90 57.76 ± 5.50	5.35 ± 2.81 6.28 ± 3.35 –	– –	– –	Age, gender, years of education	–
2	Gou/2018	DPD NDPD HC	MDS‐UPDRS‐III	GDS‐15	28/17 56/36 37/21	61.43 ± 10.06 63.97 ± 8.31 60.35 ± 11.70	– – –	None None –	– – –	Age, sex, MoCA scores, education, UPDRS‐III scores, H&Y stage, the side of onset	GDS‐15 scores

*Note*: –, data not reported.

Abbreviations: BDI, Beck's Depression Inventory; DPD, depressed Parkinson's disease patients; DSM, Diagnostic and Statistical Manual of Mental Disorders; GDS, Geriatric Depression Scale; H&Y, Hoehn and Yahr scale; HC, healthy controls; HDRS or HAMD, Hamilton Depression Rating Scale; LARS, Lille Apathy Rating Scale; LEDD, levodopa equivalent doses; MDS, Movement Disorder Society; MoCA, Montreal Cognitive Assessment; NDPD, nondepressed Parkinson's disease patients; PD, Parkinson's disease patients; RBD, Rapid eye movement sleep behavior disorder; SAS, Starkstein Apathy Scale; UKPDSBB, United Kingdom Parkinson's Disease Society Brain Bank; UPDRS, Unified Parkinson's Disease Rating Scale.

^a^ Mentioned only if the whole number of participants did not undergo DTI evaluation.

Most of the included studies did not report any information regarding the type or duration of PD or depression medication treatments (Ansari et al., 2019; Ghazi Sherbaf et al., 2018; Hu et al., 2020; Matsui et al., 2007; Prange et al., 2019; Won et al., 2019); however, three studies specified no anti‐depressant treatment among their participants (Gou et al., 2018; Huang et al., 2014; Li et al., 2020). Regarding PD symptom scores, three studies did not provide Hoehn and Yahr (H&Y) scale scores (Huang et al., 2014; Li et al., 2010; Won et al., 2019). Nevertheless, Unified Parkinson's Disease Rating Scale (UPDRS‐III) scores were reported in all studies except for the study by Ansari et al. (Ansari et al., 2019). Montreal Cognitive Assessment (MoCA) or Mini‐mental state exam (MMSE) scores were reported in all included studies, except for two (Hu et al., 2020; Prange et al., 2019; Table 2).

**TABLE 2:** Overview of included articles: Cognitive findings and correspondence of diffusion/network parameters with depression and related findings

Study		Study groups	Mental status in PD versus HC	Association between diffusivity/network metrics and other clinical findings of PD‐dep	H&Y	UPDRS‐III	MoCA or MMSE/MoCa	Depression scale	Association between diffusivity/network metrics and tests of PD‐dep
1	Li/2020	DPD NDPD HC	No differences in MMSE scores were identified between DPD patients, HC and NDPD (*p* > 0.05).	–	1.62 ± 0.64 1.78 ± 0.59 –	26.20 ± 11.71 25.85 ± 13.26 –	28.43 ± 1.25 28.57 ± 1.73 28.85 ± 2.30 (MMSE)	21.87 ± 6.47 7.02 ± 3.17 3.06 ± 3.65(HDRS)	–
2	Won/2019	DPD NDPD	–	–	– –	34.72 ± 16.33 33.82 ± 14.04	27.06 ± 1.80 27.16 ± 2.54(MoCA)	6.86 ± 1.12 4.58 ± 0.75 (GDS)	Strong correlation with GDS scores.
3	Prange/2019	Apathetic Parkinson patients Non‐apathetic PD patients HC	–	Decreased FA in the right substantia nigra and posterior putamen (focal) in PD, regardless of associated apathy.	– – –	31.5 29 –	– – –	15/−17/17 5/−28/7 4/−30/6 BDI‐II/LARS/Starkstein scores in order	–
4	Lacey/2019	DPD NDPD			1.89 ± 0.47 1.74 ± 0.45	27.78 ± 14.55 23.11 ± 10.25	27.28 ± 1.81 27.40 ± 2.17 (MoCA)	7.0 ± 2.45 11.13 ± 2.31 (GDS)	–
5	Ansari/2018	DPD NDPD			1.23 ± 0.48 1.44 ± 0.51	– –	27.69 ± 1.73 27.77 ± 2.48 (MoCA)	5.2 ± 0.04 3.16 ± 1.09 (GDS)	–
6	Ghazi Sherbaf/2018	Depression +/RBD+PD Depression +/RBD−PD Depression −/RBD–PD Depression −/RBD+PD HC	–	–	1.8 ± 0.4 1.4 ± 0.5 1.7 ± 0.5 1.5 ± 0.5	21.7 ± 8.3 19.3 ± 7.3 24.7 ± 8.7 21.8 ± 11.4	27.4 ± 2.2 27.6 ± 1.8 27.6 ± 2.4 27.5 ± 1.8 (MoCA)	5.1 ± 0.9 5.2 ± 0.4 3.3 ± 1.1 3.2 ± 1.1 (GDS)	–
7	Huang/2014	DPD NDPD	–	–	2.7 ± 0.8 2.5 ± 1.0	54.6 ± 23.7 45.3 ± 26.1	26.9 ± 3.2 25.1 ± 5.7 (MMSE)	19.9 ± 7.4 2.3 ± 1.8 (HDRS)	Negative correlation between FA values in deep temporal cortex of DPD and HDRS score
8	Li/2010	DPD NDPD	–	–	1.96 ± 0.99 1.83 ± 0.75	39.04 ± 22.28 33.83 ± 15.09	29.5 ± 0.7 29.2 ± 1.0 (MMSE)	15.64 ± 4.21 4.44 ± 2.14 (HAMD)	No correlation with HAMD for all PD patients. Negative correlation between HAMD scores and FA values in the detected right and left mediodorsal thalami for DPD and NDPD
9	Matsui/2007	DPD NDPD	–	–	3.1 ± 0.4 3.1 ± 0.4	34.9 ± 14.7 27.4 ± 14.1	25.6 ± 4.5 27.2 ± 3.3 (MMSE)	16.9 ± 7.4 6.4 ± 1.8 (HAMD)	–
1	Hu/2020	DPD NDPD HC	–	–	1.43 ± 0.59 1.63 ± 0.54 –	27.65 ± 13.17 26.21 ± 13.44 –	– – –	20.45 ± 4.58 6.98 ± 3.29 2.17 ± 2.42 (HDRS)	–
2	Gou/2018	DPD NDPD HC	–	–	– – –	25.32 ± 9.08 22.41 ± 9.51 –	27.71 ± 1.76 27.7 ± 2.05 28.24 ± 1.23 (MoCA)	7.07 ± 2.35 1.39 ± 1.29 –(GDS)	No significant correlations between FA/MD and GDS scores All node topological properties had a negative correlation with GDS scores Thirteen nodes were associated with depressive symptoms in PD patients The largest subnetwork, which the edge connective strengths were negatively associated with GDS‐15 scores, and 12 edges which connected these nodes –No subnetworks with edge strength were found to be associated with GDS‐15 scores positively

*Note*: –, data not reported.

Abbreviations: BDI‐II, Beck Depression Inventory II; DPD, depressed Parkinson's disease patients; GDS, Geriatric Depression Scale; H&Y, Hoehn and Yahr scale; HAMD‐HDRS, Hamilton Depression Rating Scale; HC, Healthy controls; LARS, Lille Apathy Rating Scale; MDS‐UPDRS, Movement Disorder Society‐Sponsored Revision of the Unified Parkinson's Disease Rating Scale; MMSE, Mini mental state exam; MoCA, Montreal Cognitive Assessment; NDPD, nondepressed Parkinson's disease patients; PD, Parkinson's disease patients; UPDRS‐III, Unified Parkinson's Disease Rating Scale Part 3 (motor assessment).

Only one study reported morphometric data in addition to DTI data (Li et al., 2020). All included studies described abnormalities observed in the brain microstructure or network properties of the brain. Tractography, a 3D reconstruction method to evaluate neural tracts using DTI data (Berman et al., 2008), was the most used analysis method. Other applied methods included region of interest (ROI), tract‐based spatial statistics (TBSS), a voxel‐based hypothesis‐free technique that utilizes FA but can only be applied to WM, and voxel‐based analysis (VBA) that contrasts the DTI data in each voxel to a template and, unlike TBSS, is applicable to GM and WM. The DTI measures assessed in the included studies are detailed in (Figure 2). Most all studies analyzed the whole brain or multiple major WM regions or tracts, whereas Li et al. analyzed only the thalamus region (Li et al., 2010) (Table 3).

**TABLE 3:** Overview of reviewed articles; DTI analysis and between‐group diffusion findings

Study		Diffusion studies							
			DTI analysis		Between‐groups findings				
		Study groups	Field Strength/b value (s/mm^2^)	Method of analysis: studied tracts/regions	FA	MD	AD	RD	Other imaging findings
1	Li/2020	DPD NDPD HC	3.0 T/1000	ROI: body of corpus callosum, splenium of corpus callosum, fornix column and body, right retrolenticular, superior corona radiate, right anterior corona radiate, external capsule, part of left internal capsule, right cingulum, left hippocampus, right ornix (cres), left stria terminalis, superior fronto‐occipital fasciculus, inferior fronto‐occipital fasciculus, right uncinate, left fasciculus	No significant difference was seen between groups (*p* > .05, FDR corrected).	Higher MD values between DPD and HC in different brain regions such as bilateral fornix(cres)/bilateral inferior front occipital fasciculus, stria terminalis, the body of corpus callosum, left uncinated fasciculus, splenium of corpus callosum, bilateral superior corona radiata, bilateral external capsule, bilateral superior fronto‐occipital fasciculus, right hippocampal part of cingulum and right uncinate fasciculus (All had *p* < .05, FDR corrected).	Higher AD values between DPD and HC in several brain regions including the right superior fronto‐occipital fasciculus, bilateral retrolenticular part of internal capsule, splenium of corpus callosum, bilateral external capsule, bilateral superior corona radiata, right cingulated gyrus, right hippocampal part of cingulum, bilateral superior longitudinal fasciculus, bilateral fornix(cres)/stria terminalis, left superior fronto‐occipital fasciculus, bilateral inferior fronto‐occipital fasciculus, and bilateral uncinate fasciculus(All had *p* < .05, FDR corrected).	No significant difference was seen between groups. (*p* > .05, FDR corrected).	No significant differences were observed between groups in WMV (*p* > .05, FDR corrected). When compared to NDPD and HC, DPD showed microstructural impairment in the left hippocampal part of the cingulum, right anterior corona radiates, and the body of the corpus callosum. DPD showed damage to the microstructure of white matter that was mainly located in the frontallimbic regions and hippocampus, which were associated with mood adjustments.
2	Won/2019	DPD HC	3.0 T/1000	Probalistic tractography between ROIs: right hippocampus, left anterior cingulate and paracingulate gyri, amygdala, left postcentral gyrus, left precuneus, temporal pole: left superior temporal gyrus, left middle temporal gyrus	–	–	–	–	Larger degree centrality of structural connectivity in DPD versus NDPD in hippocampus, amygdala, postcentral gyrus, and temporal pole
3	Prange/2019	Apathetic Parkinson patients Non‐apathetic PD patients HC	1.5 T/1000	TBSS: left subcallosal gyrus and inferior frontal gyrus, medial frontal gyrus, left globus pallidus (internal and external), left subcallosal gyrus and medial frontal gyrus, left caudate nucleus (head and body), cingulate gyrus, caudate nucleus (head and body), pregenual anterior cingulate cortex, right uncus, cerebellum (posterior lobe), midbrain	Apathetic patients showed great microstructural anomalies with reduced FA in the bilateral medial thalamus. The greater the apathy the more considerable decrease in FA in the medial frontal, subcallosal gyrus, subgenual anterior cingulate cortices, and anterior striatum.	Apathetic patients showed great microstructural anomalies with increased MD in the bilateral medial thalamus.	–	–	No statistically significant difference was observed in gray‐matter microstructure in the limbic system. MO rose with apathy severity in the caudal midbrain, adjoining the morphological deformation observed on the midline, at the level of the decussation of the superior cerebellar peduncles
4	Lacey/2019	DPD NDPD	3.0 T/1000	TBSS: Whole brain ROI: uncinate fasciculus, thalamic radiation, longitudinal fasciculus, forceps minor	No differences in white matter integrity in specific ROIs or in the whole brain level.	No differences in white matter integrity in specific ROIs or in the whole brain level.	No differences in white matter integrity in specific ROIs or in the whole brain level.	No differences in white matter integrity in specific ROIs or in the whole brain level.	–
5	Ansari/2018	DPD Non‐depressed NDPD	3.0 T/1000	Deterministic fiber tracking: left and right uncinate fasciculi, left and right inferior longitudinal fasciculi, left and right fornices, left inferior fronto–occipital fasciculus, right corticospinal tract, genu of corpus callosum, middle cerebellar peduncle	–	–	–	–	A compelling difference (FDR = 0.016129) was seen in the left and right uncinat fasciculi (UF),left and right inferior longitudinal fasciculi (ILF), left and right fornices, left inferior fronto‐occipital fasciculus (IFOF), right corticospinal tract, genu of corpus callosum (gCC), and middle cerebellar peduncle. Meaning, depressive disorders were corelated with decreased connectivity in the regions mentioned above
6	Ghazi Sherbaf/2018	Depression +/RBD+PD Depression +/RBD−PD Depression −/RBD–PD Depression −/RBD+PD HC	3.0 T/1000	Deterministic tractograghy: Bilateral superior longitudinal fasciculus, left inferior longitudinal fasciculus, body of corpus callosum, bilateral U‐fiber, bilateral and right cingulum	–	–	–	–	Decreased connectivity in B‐SLF, B‐U‐fiber, left inferior longitudinal fasciculus (L‐ILF), body of CC was observed between depression +/RBD+ PD and HC Decreased connectivity in B‐SLF, L‐U‐fiber, L‐ILF, B‐cingulum between depression +/RBD−PD and HC Decreased connectivity in the genu, splenium and body of CC, bilateral cingulum, left ILF, left fornix, right SCP, and right inferior fronto‐occipital fasciculus (IFOF) between depression +/RBD+ PD and depression –/RBD–PD. Lower connectivity in the right cingulum, left ILF, splenium, and body of the CC between depression +/RBD+ PD and depression −/RBD+PD Lower connectivity in the bilateral cingulum, bilateral fornix, bilateral ILF, left UF, right CST and genu, splenium, and body of CC between depression +/RBD−PD and depression −/RBD−PD
7	Huang/2014	DPD NDPD	3.0 T/1000	TBSS: Whole brain	Decreased FA in several brain regions in the DPD group. Damaged fibers were located in the left uncinate fasciculus (UF), the left superior longitudinal fasciculus (SLF), left anterior thalamic radiation, left forceps minor and the inferior longitudinal fasciculus.	No significant difference was seen between groups.	–	–	–
8	Li/2010	DPD NDPD	3.0 T/1000	VBA: Whole thalamus	Significant decrease was identified in thalamus in 2 regions: right and left mediodorsal thalami. No increase was observed in any of the regions.	No significant difference was seen between groups.	–	–	–
9	Matsui/2007	DPD NDPD	1.5 T/1000	ROI: orbitofrontal white matter, prefrontal white matter, temporal white matter, occipital white matter, parietal white matter, anterior cingulate bundle, posterior cingulate bundle	Significant decrease in FA values in the frontal ROIs possibly representing bilateral anterior cingulate bundles in patients with depression. No differences were observed in FA values in other regions, including the orbitofrontal and prefrontal white matter.	–	–	–	–

Network studies											
Study		Network analysis	Between‐groups findings (PD vs. HC)								
		Field Strength/b value (s/mm^2^)	Method of analysis	Global Parameters						Nodal parameters	Other imaging findings
				Clustering coefficient (*γ*)	local efficiency	Betweenness centrality	Shortest path length (*λ*)	Global efficiency *p*	Small‐worldness (σ:*γ*/*λ*)		
1	Hu/2020	3.0 T/1000	Deterministic tractography: left middle frontal gyrus, left middle occipital gyrus, right precuneus, right fusiform gyrus, right inferior temporal gyrus, bilateral middle temporal gyri, bilateral supplementary motor areas, bilateral precentral gyri, bilateral median cingulate and paracingulate gyri, heschl gyrus, right orbital part of middle and inferior frontal gyrus, right opercular part of inferior frontal gyrus, bilateral pallidum, right putamen, left paracentral lobule, fronto‐limbic system, parahippocampal gyrus, superior medial orbital frontal gyrus, superior temporal pole, right anterior cingulate and paracingulate gyri, supramarginal gyrus	Increase (DPD vs. NDPD) Decrease (NDPD vs. HC)	Increase (DPD vs. NDPD) Decrease (NDPD vs. HC)	–	Increase (DPD vs. HC)	Decrease (DPD vs. HC)	Decrease in all groups	Decrease of the Enodal in frontal, parietal, temporal and subcortical brain regions. (DPD&NDPD vs. HC) DPD had a decrease in efficiency when compared with HC in cortico‐subcortical circuits [right pallidum and putamen of lenticular nucleus, left paracentral lobule, heschl gyrus]; frontolimbic system [left hippocampus and parahippocampal gyrus, right middle frontal gyrus, superior temporal gyrus, dorsolateral superior frontal gyrus, insula, rolandic operculum, right orbital part of middle and inferior frontal gyrus, right opercular part of inferior frontal gyrus]; default mode systems [right posterior cingulate gyrus and right precuneus]; and an increase of efficiency in the left supramarginal gyrus, right orbital part of the superior frontal gyrus, post central gyrus. NDPD had a decrease in efficiency compared with HC in cortico‐subcortical circuits [bilateral pallidum, right putamen, left paracentral lobule, left supplementary motor area]; fronto‐limbic system [left hippocampus, parahippocampal gyrus, superior medial orbital frontal gyrus, superior temporal pole, dorsolateral part of the superior frontal gyrus]; and an increased efficiency in right anterior cingulate and paracingulate gyri, supramarginal gyrus, left insula and left rolandic operculum. DPD group had a higher Enodal compared with NDPD [*p* < 0.05, uncorrected] mainly in frontolimbic system (right orbital part of the inferior frontal gyrus, left medial orbital part of the superior frontal gyrus, right opercular part of the inferior frontal gyrus, right insula, bilateral rolandic operculum, the left posterior cingulate gyrus) and left superior temporal gyrus.	–
2	Gou/2018	3.0 T/1000	Deterministic tractography‐Nodes: Left hippocampus, left parahippocampus, left calcarine, left and right lingual, left superior occipital, left middle occipital, left and right inferior occipital, left fusiform, left middle temporal, left inferior temporal gyrus, right superior frontal, left putamen, left and right fusiform	–	Decrease (DPD vs. NDPD)	–	Increase (DPD vs. NDPD&HC)	Decrease (DPD vs. NDPD&HC)	Increase (NDPD vs. HC)	–	TBSS: Whole brain No significant difference in FA was observed in the DPD group compared with the NDPD group. Reduced FA was observed in forceps major, left uncinate fasciculus, left superior longitudinal fasciculus, bilateral corticospinal tract and arcuate fibers in the bilateral frontal and right occipital regions between DPD and HC. No significant difference in MD was observed in the DPD group compared with the NDPD group. Increased MD was seen when comparing DPD with HC in several regions, including the genu of the corpus callosum, the body and column of the fornix, the left crus of the fornix, bilateral corticospinal tract and arcuate fibers in the left amygdala, left insula, bilateral frontal, bilateral parietal, bilateral temporal and right occipital regions.

*Note*: –, data not reported.

Abbreviations: AD, axial diffusivity; DPD, depressed Parkinson's disease patients; FA, fractional anisotropy; HC, healthy controls; LP, characteristic path length; MD, mean diffusivity; MO, mode of anisotropy; NDPD, nondepressed Parkinson's disease patients; PD, Parkinson's disease patients; RD, radial diffusivity; ROI, region of interest; TBSS, Tract based spatial statistics; VBA, voxel based analysis; WMV, White matter volume.

Besides comparisons made between DPD and NDPD patients, all included studies except Prange et al. (2019), also made comparisons with other diagnostic groups, including three studies that compared DPD patients to HC (Ghazi Sherbaf et al., 2018; Hu et al., 2020; Li et al., 2020), one study that compared apathetic PD versus non‐apathetic PD cases and apathetic PD versus HC cases (Prange et al., 2019), and one study that compared DPD with rapid eye movement sleep behavior disorder(RBD) versus DPD without RBD (Ghazi Sherbaf et al., 2018; Table 1). Moreover, four studies (Gou et al., 2018; Huang et al., 2014; Li et al., 2010; Won et al., 2019) specifically assessed correlations between diffusivity or network measures with H&Y, UPDRS, and GDS measures of DPD (Table 2).

### 3.2 White matter microstructural/network alterations

#### 3.2.1 Overview

As illustrated in Table 2, studies using ROI analysis showed no significant difference in FA measures between groups (Lacey et al., 2019; Li et al., 2020); however, the study of Matsui et al. revealed a significant FA decrease in the frontal ROIs (Matsui et al., 2007). Similarly, both increased MD and AD values (Li et al., 2020) or no between study subject differences (Lacey et al., 2019) have been reported. Additionally, two studies reported no differences in RD between DPD and NDPD groups (Lacey et al., 2019; Li et al., 2020). TBSS analysis revealed no AD and RD groups differences (Lacey et al., 2019), and decreased FA (Prange et al., 2019; Gou et al., 2018) (the latter comparing DPD patients and HC) as well as negligible FA differences in the studies by Gou et al. (2018), Lacey et al. (2019) (between DPD patients and NDPD subjects). Increased MD was reported in some studies comparing DPD patients versus HC (Gou et al., 2018; Prange et al., 2019) but not others (Huang et al., 2014; Lacey et al., 2019). One study that performed voxel‐based analysis revealed lower FA values between DPD patients and NDPD patients and no MD differences (Li et al., 2010). All studies reporting alterations in DTI measures observed widespread differences with the involvement of both hemispheres. Additionally, alterations of white matter volume (WMV) between DPD, HC, and NDPD subjects, GM microstructure and mode of anisotropy between apathetic PD, non‐apathetic PD, and HC, and degree centrality changes between DPD and HC have been reported in several studies (Li et al., 2020; Prange et al., 2019; Won et al., 2019).

**FIGURE 3:**
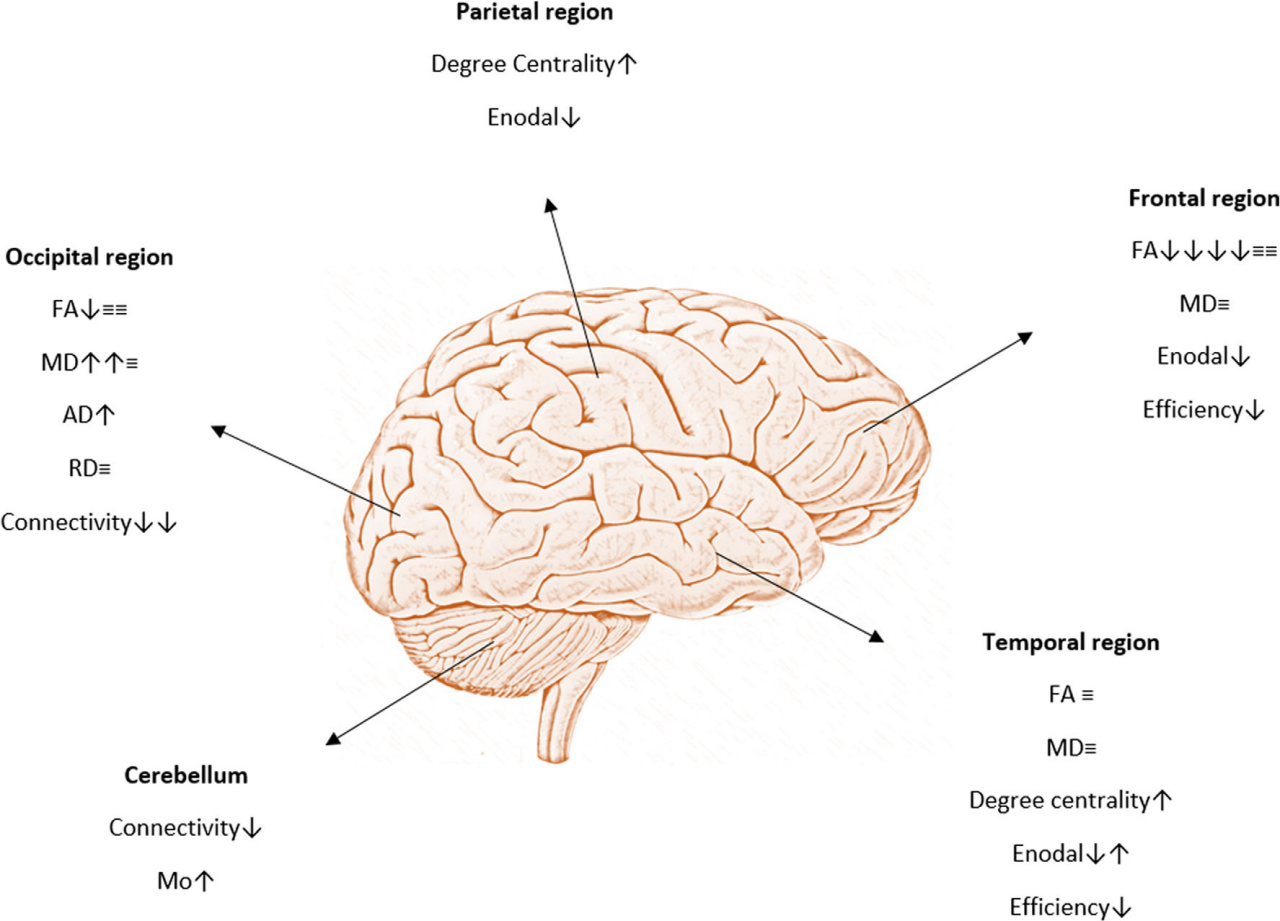
The most affected cortical regions in DPD patients when compared to NDPD/healthy subjects. Each symbol summarizes findings from an included study. Upward arrow: increase; downward arrow: decrease; ≡: no significant change

Two studies conducting deterministic tractography analyses demonstrated alterations in network integration variables, mainly a significant increase in clustering coefficient (*γ*) and shortest path length (*λ*) and a significant decrease in global efficiency in DPD compared to NDPD and HC (Gou et al., 2018; Hu et al., 2020). Those findings suggest a higher capability of a local combination of data and slower interactional speeds between brain regions with a reduced capacity to process information. (Figure 3).

As noted above, Hu et al. reported an increase in the clustering coefficient (*γ*) and local efficiency in DPD compared to NDPD patients (Hu et al., 2020). However, NDPD patients showed a decrease in both parameters compared to HC. Although small worldness (*σ*:*γ*/*λ*) was decreased in all PD groups compared to NDPD and HC in the study by Hu et al. (Hu et al., 2020), Gou et al. found this to be increased in NDPD compared to HC subjects (Gou et al., 2018). Betweenness centrality was not reported by any of the network studies.

#### 3.2.2 Between‐group contrasts: brain lobes

Alterations in diffusivity in the frontal, temporal and parietal regions were demonstrated in four studies comparing DPD and NDPD subjects, including two network studies. Probabilistic tractography between the postcentral gyrus, the right hippocampus, and the anterior end of the temporal lobe, the temporal pole, revealed a significant increase in degree centrality of structural connectivity in DPD patients compared to NDPD subjects (Won et al., 2019). ROI analysis in the study of Li et al. revealed a significant increase in the index of axonal integrity in the right cingulate gyrus but no significant differences in AD and RD for both the right cingulate gyrus and the left hippocampus between diagnostic groups (Li et al., 2020). This study also showed that microstructural impairments could be seen in the fronto‐limbic and hippocampus regions which are associated with mood regulation. TBSS analysis by Prange et al. revealed that greater apathy in DPD subjects was correlated with a significant decrease in FA in the medial frontal gyrus (Prange et al., 2019). However, no statistically significant differences were observed in the microstructure of the limbic system GM. On the contrary, Gou et al. reported that TBSS analyses of the whole brain revealed no differences in FA and MD values comparing DPD patients with NDPD subjects (Gou et al., 2018).

Matsui et al. demonstrated decreased anisotropy in the frontal ROIs of DPD patients (Matsui et al., 2007). This was also observed in network studies, where deterministic tractography showed a decrease of the nodal efficiency (Enodal) in the frontal, temporal, and with less preponderance, the parietal lobes when comparing DPD patients, NDPD subjects, and HC. While DPD subjects had increased network efficiency compared to HC subjects in the left supramarginal gyrus, right orbital part of the superior frontal gyrus, and the right posterior central gyrus, they showed decreased network efficiency in the frontal gyrus, the DMN (right posterior cingulate gyrus and right precuneus), and the fronto‐limbic system (left hippocampus and parahippocampal gyrus, right middle frontal gyrus, superior temporal gyrus, dorsolateral superior frontal gyrus, insula, rolandic operculum, right orbital part of middle and inferior frontal gyrus, right opercular part of inferior frontal gyrus). NDPD patients also demonstrated decreased efficiency when compared to HC in the left paracentral lobule, frontal gyrus, and the fronto‐limbic system (left hippocampus, parahippocampal gyrus, superior medial orbital frontal gyrus, superior temporal pole, dorsolateral part of the superior frontal gyrus). However, increased efficiency was observed in the cingulate and paracingulate gyri, the left insula, the right supramarginal gyrus, and the left rolandic operculum. When comparing DPD patients with NDPD subjects, Enodal was higher in DPD patients mainly in the left superior temporal gyrus and the fronto‐limbic system, which consists of the right orbital part of the inferior frontal gyrus, left medial orbital part of the superior frontal gyrus, right opercular part of the inferior frontal gyrus, right insula, bilateral rolandic operculum, and the left posterior cingulate gyrus (Hu et al., 2020).

#### 3.2.3 Between‐group contrasts: white matter

Diffusivity of the main association fibers connecting the major lobes and regions, such as the frontal, temporal, and, to a less extent, the parietal regions, was altered in DPD patients. This can be observed in multiple ROI (Lacey et al., 2019; Li et al., 2020; Matsui et al., 2007), TBSS (Huang et al., 2014; Lacey et al., 2019; Prange et al., 2019), and tractography (Ansari et al., 2019; Ghazi Sherbaf et al., 2018; Gou et al., 2018) analysis methods.

Comparing FA measures in DPD patients and NDPD subjects, varying results were found within the studies of Li et al., Lacey et al. and Gou et al., finding no evidence of differences between WM clusters, such as the uncinate fasciculus, forceps minor, left and right lingual, and bilateral fusiform (Gou et al., 2018; Lacey et al., 2019; Li et al., 2020) or for whole‐brain (Gou et al., 2018; Lacey et al., 2019). Conversely, Huang et al. reported decreased FA in DPD patients with alterations mainly to the left uncinate fasciculus, left superior longitudinal fasciculus, left anterior thalamic radiation, left forceps minor, and the inferior longitudinal fasciculus (Huang et al., 2014). Matsui et al. also reported decreased FA in DPD patients, but only for the bilateral anterior cingulate bundle with no differences observed in the orbitofrontal and prefrontal WM regions (Matsui et al., 2007). Two studies compared WM FA measures between DPD subjects and HC, one of which (Li et al., 2020) observed no differences while the other (Gou et al., 2018) demonstrated decreased WM FA in DPD patients. Prange et al. described that more apathetic PD patients had lower FA (Prange et al., 2019).

Assessing the relative mobility of water molecules, DPD patients exhibited increased water mobility compared to NDPD subjects across different brain regions, such as the bilateral fornix (cres)/bilateral inferior front occipital fasciculus, stria terminalis, the body of corpus callosum, bilateral corticospinal tract, and arcuate fibers in the left amygdala, when compared to HC in two studies (Gou et al., 2018; Li et al., 2020) but no evidence of differences compared to NDPD subjects in the uncinate fasciculus, longitudinal fasciculus, forceps minor (Lacey et al., 2019) or for whole‐brain (Gou et al., 2018; Huang et al., 2014; Lacey et al., 2019).

The axonal and myelin integrity indices did not differ between DPD and HC subjects (Li et al., 2020) or between DPD and NDPD subjects (Lacey et al., 2019), although the study by Li et al. showed higher AD values between DPD and HC subjects (Li et al., 2020). Decreased connectivity for DPD participants in the inferior longitudinal fasciculus and the corpus callosum was reported in two studies (Ansari et al., 2019; Ghazi Sherbaf et al., 2018; Table 3).

#### 3.2.4 Between‐group contrasts: subcortex

Several studies have reported damage to subcortical structures of the brain, including the thalamus that is considered to be a major neural gateway connecting the medial temporal, frontal lobe, and the parieto‐occipital regions via the anterior–posterior thalamic radiations, respectively (Sanjari Moghaddam et al., 2019). ROI analysis of the amygdala and thalamic radiations showed more significant degree centrality of structural connectivity with the amygdala, revealing no evidence of a statistical difference in WM FA, MD, RD, or AD values of the anterior and posterior thalamic radiations in association with DPD patients (Lacey et al., 2019; Won et al., 2019). On the contrary, TBSS analytics revealed microstructural anomalies in the bilateral medial thalamus with significantly increased MD and reduced FA in one study (Prange et al., 2019). TBSS analysis in another study showed decreased FA in the left anterior thalamic radiation, though no evidence was found for MD differences comparing DPD and NDPD subjects (Huang et al., 2014).

The only reported study that employed voxel‐based analyses (Li et al., 2010) evaluated the whole thalamus and identified decreased FA in both the right and left mediodorsal thalami in DPD versus NDPD subjects. MD, however, had no significant difference between the study groups. Among the included network studies, Hu et al. reported that DPD patients, similar to NDPD subjects, had decreased efficiency in cortico‐subcortical circuits, that consisted of the right pallidum, and putamen, lenticular nucleus, the left paracentral lobule, and the Heschl's gyrus when compared to HC (Hu et al., 2020).

#### 3.2.5 Between‐group contrasts: cerebellum

Very few of the included studies reported possible microstructural anomalies in the cerebellum of DPD patients, and, among those studies, only the superior and middle cerebellar peduncles were assessed. By using deterministic fiber tracking, Ansari et al. showed that depression in PD patients was correlated with significantly decreased connectivity in the cerebellum, more specifically the middle cerebellar peduncle (Ansari et al., 2019). TBSS analysis also detected midline morphological deformation at the level of decussation of the superior cerebellar peduncle (Prange et al., 2019).

#### 3.2.6 Correlations between diffusivity/network metrics and cognitive status

Two cognitive assessments of PD subjects, the MMSE and MoCA, were carried out in almost all included studies, except the study by Prange et al. In these studies, both the mean MMSE and mean MoCA scores were higher than 25 in DPD patients, which may suggest normal cognitive functioning. It is important to note that Hu et al. did not mention any values for the participants' MMSE scores and that the studies of Li et al. and Hu et al. excluded PD subjects with an MMSE score of <24 (Hu et al., 2020; Li et al., 2020). Li et al. found no evidence of MMSE score differences between DPD patients, NDPD patients, and HC (Li et al., 2020). None of the other studies reported any correlations between microstructural abnormalities and cognitive function measures (Table 2).

#### 3.2.7 Correlations between diffusivity/network metrics and PD duration/mood severity

Sanjari Moghaddam et al. (2020) has reported an association between PD disease duration and network connectivity of several cortical regions and the WM structural network. However, the seven included studies that detailed the disease duration of DPD patients did not report that disease duration was correlated with microstructural anomalies (Ghazi Sherbaf et al., 2018; Hu et al., 2020; Huang et al., 2014; Lacey et al., 2019; Li et al., 2010, 2020; Matsui et al., 2007).

PD disability assessments, including the H&Y and UPDRS, were conducted on most study subjects. However, PD disability scores were not reported to have any association with diffusivity/network metrics. GDS is a clinical score representing the degree of depression. Patients with a GDS score of 6 or more are regarded as depressive subjects (Yesavage et al., 1982). Won et al. showed a strong correlation between the GDS score and selected imaging features (Won et al., 2019). One of the network studies, Gou et al. (2018), found no evidence of correlations between FA/MD values and GDS scores. On the other hand, a negative association was reported between GDS scores and all of its node topological properties and a large subnetwork with 12 edges connecting its nodes, including the left hippocampus, left parahippocampus, left calcarine, left lingual, left superior occipital, left middle occipital, left inferior occipital, left fusiform, left middle temporal and left inferior temporal gyrus (Gou et al., 2018).

HDRS, also referred to as the HAMD, is a questionnaire that is used to assess depression severity, tapping both mood and somatic symptoms, and also is commonly used as a measure of recovery in treatment trials or naturalistically over time (Biegler, 2018). Huang et al. (2014) reported a negative correlation between HDRS scores and FA values in the deep temporal cortex. HDRS was also reported to be negatively correlated with FA values in the bilateral mediodorsal thalami in both DPD patients and NDPD subjects (Li et al., 2010; Table 2).

One study did find that increasing depression symptom severity was positively correlated with focal FA reduction, affecting mainly the right substantia nigra and posterior putamen (Prange et al., 2019). No other studies reported any associations between diffusivity/network metrics and other clinical features of DPD patients.

## 4 DISCUSSION

In summary, DTI studies included in this study reported that DPD patients may exhibit brain microstructural anomalies compared to NDPD or HC subjects. While the affected tracts and regions may differ between studies, several areas, that include the fronto‐temporal lobes, fronto‐limbic, hippocampus, thalamus and its radiations, cerebellum, and WM clusters, such as the uncinated fasciculus and the corpus callosum, are more consistently implicated. Many studies have demonstrated that alterations of these regions can substantially impact cognition and awareness and may underlie the cognitive impairments observed in previous studies of PD patients (Verbaan et al., 2007; Wolff & Vann, 2019).

In contrast to Sanjari Moghaddam et al. (2020), studies abstracted in the current review indicate no relation between disease duration and DTI abnormalities. As age, disease onset, and development of depression in PD cases, as well as medications effects, may influence disease duration effects on microstructural abnormalities, further studies that include larger, more clinically characteristic samples may help to clarify this relationship. Regarding the severity of depression, the reported studies, albeit with mixed results and associations, still collectively suggest that the severity of depressive symptoms in DPD patients may affect the extent of WM microstructures alterations in different brain regions. Prior studies that have concluded that patients with depressive disorders may show microstructural disconnectivity, particularly in the corpus callosum and corona radiate (van Velzen et al., 2020), are consistent with the present findings. It should also be noted that many factors can limit efforts to employ neuroimaging modalities to diagnose depression in a particular PD patient, namely because of the considerable symptom variation among PD patients having the same diagnosis, substantially different signs, and symptoms that depression may produce in individuals, and finally the possibility of overlapping brain regions that may be affected by other comorbid psychiatric conditions.

The included papers reported mixed DTI results. The study by Lacey et al. revealed no DTI differences between DPD and NDPD subjects for specific ROIs (Lacey et al., 2019), which does not support the reproducibility of findings presented by Huang et al. (2014). This may be due to the small number of participants in each study, showing the need for larger subject groups having the full expression of PD characteristics, including cognitive impairment. However, Gou et al. (2018) also found similar overlapping regional findings as Huang et al. (2014) in the uncinate and longitudinal fasciculus, suggesting damage to the limbic system and its functions (Gou et al., 2018; Huang et al., 2014). The network studies demonstrated an increase in characteristic path length and reduction in global efficiency, which can be conceptualized as a breakdown of brain network unity (Gou et al., 2018; Hu et al., 2020). Consistent with this, an fMRI study by Quin et al. also showed an increase in the characteristic path length of the functional network in DPD subjects compared to HC (Qian et al., 2017). These alterations suggest lower efficiency and additional costs of data transfer between different functional areas in DPD patients. Impaired brain network data transmission could result in, or amplify, motor retardation, a prominent somatic symptom both of depression and PD.

The study by Lacey et al. (2019) revealed no significant WM microstructural differences in the thalamic region among DPD patients. In contrast, two other studies did find alterations in the thalamus and its radiations in DPD patients (Li et al., 2010; Prange et al., 2019) that is supported by studies using other neuroimaging modalities, such as fMRI, that indicate decreased connectivity in the left mediodorsal thalamus (Cardoso et al., 2009). As the thalamus processes and relays sensory information to distinct cortical regions, connections with the medial temporal lobes contribute to processing sensory input associated with learning and memory within the medial limbic circuit. This suggests an additional focus on thalamic microstructural and related network alterations in DPD patients, which may contribute to associated cognitive impairment, particularly affecting memory and attention domains.

Our study suggests that FA in WM clusters may be decreased in DPD, which indicates WM damage. However, DTI findings from several previous studies (Sanjari Moghaddam et al., 2020) do not support this relationship. In this context, we did not find that axonal and myelin integrity indices were different for DPD subjects, except for one study (Li et al., 2010). It is important to note that DTI determines microstructural WM cluster anomalies without correlation to WM volume changes, which overall do not appear to differ in PD patients compared to HC (Rektor et al., 2018). As a result, the specific relationship of myelin and axonal structure alterations related to depression in PD remains indefinite and requires further investigation.

The cerebellum is now regarded as a critical structure for cognitive and executive functions, in addition to motor coordination (Stoodley & Schmahmann, 2010). A recent fMRI resting‐state connectivity study has implicated the cerebellum as having a role in the underlying pathophysiology of depression in PD (Wang et al., 2018). As DTI studies included in this analysis mostly did not report cerebellar relationships, future DTI studies seeking to better understand microstructural abnormalities in DPD patients need to assess cerebellar and cerebro‐cerebellar networks systematically.

The studies of Li et al. and Hu et al. included cognitive indices but enrolled only subjects having mean MMSE scores higher than 24, suggesting normal cognitive functioning in the DPD patients that substantially limits conclusions regarding any interactions between regional microstructural alterations, depression, and cognition. The overall lack of cognitive impairment observed in subjects included in this analysis also makes it difficult to comment upon the interaction of PD and depression on regional microstructure alterations (Hu et al., 2020; Li et al., 2020). Thus, future studies will need to extend investigations of DPD patients to include more clinically distinct populations not selected based on an intact cognitive profile to understand better the inter‐relationships between depression and cognitive impairment from microstructural alterations. Other potentially confounding factors, such as possible structural brain differences, disease stage, age/sex differences, and concurrent medication effects, will need to be more fully addressed. Overall, 229 DPD subjects were included in this review. Thus, available studies that specifically assessed depression in PD are mostly underpowered, and the totality of DPD subjects studied is very limited, necessitating larger samples to establish more clear relationships.

As mood disturbances in PD can be difficult to disentangle from PD symptom expression, it would be necessary for future investigations to systematically evaluate whether depression associated with PD is different from the neurobiological underpinnings of major depression not associated with PD. Critical assessment of imaging findings, including DTI, evaluating differences among DPD, NDPD, individuals with major depression without PD, and HC without depression, will help better understand the neurobiological substrates of depression and PD.

The current study systematically reviewed brain network and microstructure relationships between associated depression and PD. However, this analysis has several limitations. First, DTI findings are affected by intrinsic technical factors like low anatomical resolution, low SNR and distortions (e.g., vibrational artifacts caused by mechanical deficiencies of the scanner or subject‐initiated motion during scanning), and variable capability to resolve crossing fiber tracts that restrict its diagnostic power (Solders et al., 2017). DTI findings are also subject to sequence acquisition parameters, scanner field strength, and maximum *b* values (Zhang & Burock, 2020). Thus, greater comparability across and within studies requires harmonized DTI scanning parameters, particularly for multisite studies, and standardized cutoff points for DTI indices. It is also important to note that newer, novel imaging methods and analytic techniques, having greater sensitivity and specificity, are being developed and currently applied, including fixed‐based analysis, neurite orientation dispersion and density imaging (NODDI), connectometry, and constrained spherical deconvolution (Sanjari Moghaddam et al., 2019). Further, depression is a very heterogeneous condition having a variety of signs and symptoms. In the current study, DPD subjects in the included papers exhibited an uneven distribution of depressive symptoms, which might be a source of heterogeneity and contribute to inconsistent findings.

## 5 CONCLUSION

DTI findings to date suggest altered WM microstructure and network disruptions of the cerebrum and cerebellum in PD patients with depression. Studies using various diffusion and network analytic approaches overall found alterations to essential brain structural networks, including impaired network integrity for specific cortical regions, such as the temporal and frontal cortices. Additionally, findings indicate that microstructural changes in specific limbic structures, such as the prefronto‐temporal regions and connecting WM pathways, are altered in DPD compared to NDPD. There remain inconsistencies between studies reporting DTI measures, such as FA and MD, and depression severity in PD participants. Additional research evaluating underlying neurobiological relationships between major depression, DPD, and NDPD is required to disentangle further mechanisms that underlie depression, and related somatic symptoms, in PD.

## CONFLICT OF INTEREST

The authors have declared no conflicts of interest for this article.

## Supporting information


**Table S1** Quality assessment of the included studies.

**Table S2** Publication bias risk assessment of the included studies.

## Data Availability

Data sharing is not applicable to this article as no new data were created or analyzed in this study.
