# Supplementary material for: Brain microstructural alterations of depression in Parkinson's disease: A systematic review of diffusion tensor imaging studies
Source: Hum Brain Mapp. 2022 Jul 20;43(18):5658–80. doi: 10.1002/hbm.26015 (PMC9704780; doi:10.1002/hbm.26015)
Supplement: Supplementary file 1 — Table S1 Quality assessment of the included studies. [file HBM-43-5658-s001.pdf]

Table S1. Quality Assessment of the included studies.

	Selection					Comparability			Exposure		
**Study First author (year)**	**Case definition adequacy**	**Representative of cases**	**Selection of controls**	**Definition of controls(healthy)**	**Subtotal**	**Age**	**Sex**	**Subtotal**	**Ascertainment of exposure**	**Non-response rate**	**Subtotal**
Li/2020	*	*		*	3	*	*	2	*	*	2
Hu/2020	*	*		*	3	*	*	2	*	*	2
Won/2019	*			*	2	*	*	2	*	*	2
Prange/2019	*	*		*	3	*		1	*	*	2
Lacey/2019	*			*	2	*	*	2	*	*	2
Ansari/2018	*			*	2			0	*	*	2
Ghazi Sherbaf/2018	*			*	2	*	*	2	*	*	2
Gou/2018	*			*	2	*	*	2	*	*	2
Huang/2014	*	*		*	3	*	*	2	*	*	2
Li/2010	*	*		*	3	*	*	2	*	*	2
Matsui/2007	*			*	2			0	*	*	2
