# Supplementary material for: Brain microstructural alterations of depression in Parkinson's disease: A systematic review of diffusion tensor imaging studies
Source: Hum Brain Mapp. 2022 Jul 20;43(18):5658–80. doi: 10.1002/hbm.26015 (PMC9704780; doi:10.1002/hbm.26015)
Supplement: Supplementary file 2 — Table S2 Publication bias risk assessment of the included studies. [file HBM-43-5658-s002.pdf]

Table S2. Publication bias risk assessment of the included studies.

Study	Selection bias		Performance bias	Attrition bias	Detection bias				Reporting bias
	**Q1**	**Q2**	**Q3**	**Q4**	**Q5**	**Q6**	**Q7**	**Q8**	**Q9**
Li/2020	Yes	Yes	Yes	Yes	No	Yes	Yes	Yes	Yes
Hu/2020	Yes	Yes	Yes	Yes	No	Yes	Yes	Yes	Yes
Won/2019	No	Yes	Yes	Yes	No	Yes	Yes	Yes	Yes
Prange/2019	Yes	Yes	Yes	Yes	No	Yes	Yes	Yes	Yes
Lacey/2019	No	Yes	Yes	Yes	No	Yes	Yes	Yes	Yes
Ansari/2018	No	No	Yes	Yes	No	Yes	Yes	No	Yes
Ghazi Sherbaf/2018	No	Yes	Yes	Yes	No	Yes	Yes	Yes	Yes
Gou/2018	Yes	Yes	Yes	Yes	No	Yes	Yes	Yes	Yes
Huang/2014	Yes	Yes	Yes	Yes	No	Yes	Yes	Yes	Yes
Li/2010	Yes	Yes	Yes	Yes	No	Yes	Yes	Yes	Yes
Matsui/2007	Yes	Yes	Yes	Yes	No	Yes	Yes	Yes	Yes
